# Supplementary material for: Murepavadin Enhances the Killing Efficacy of Ciprofloxacin against Pseudomonas aeruginosa by Inhibiting Drug Efflux
Source: Antibiotics (Basel). 2024 Aug 26;13(9):810. doi: 10.3390/antibiotics13090810 (PMC11429200; doi:10.3390/antibiotics13090810)
Supplement: Supplementary file 1 [file antibiotics-13-00810-s001.zip › antibiotics-3125935-supplementary.pdf]

**Table S1.** Bacterial strains, plasmids and primers used in this study

| Strain | Description | Source |
|---|---|---|
| *P. aeruginosa* | | |
| PA14 | Wild type strain of *P. aeruginosa* | [59] |
| CI-PA-25 | Clinical isolate | This study |
| CI-PA-26 | Clinical isolate | This study |
| CI-PA-42 | Clinical isolate | This study |
| CI-PA-45 | Clinical isolate | This study |
| CI-PA-82 | Clinical isolate | This study |
| CI-PA-83 | Clinical isolate | This study |
| CI-PA-92 | Clinical isolate | This study |
| CI-PA-123 | Clinical isolate | This study |
| CI-PA-128 | Clinical isolate | This study |
| CI-PA-132 | Clinical isolate | This study |
| CI-PA-139 | Clinical isolate | This study |
| CI-PA-161 | Clinical isolate | This study |
| Primers | Sequence 5’-3’ | Function |
| *rpsL*-RT-S | CAAGCGCATGGTCGACAAGAG | RT-PCR |
| *rpsL*-RT-AS | ACCTTACGCAGTGCCGAGTTC | RT-PCR |
| *PA0614*-RT-S | CGCTGCCTGCCAAGGA | RT-PCR |
| *PA0614*-RT-AS | ATCAGTACCCAGAGCGGCATT | RT-PCR |
| *PA0629*-RT-S | GCCATGGACGAGAGGGAGAT | RT-PCR |
| *PA0629*-RT-AS | CCTTGGGCGCTGTAATTGAG | RT-PCR |
| *recA*-RT-S | TGAAGTTCTACGCCTCGGTC | RT-PCR |
| *recA*-RT-AS | GAAACCTTGTTCTTCACCAC | RT-PCR |

**Table S2.** MICs (mg/L) of indicated antibiotics for PA14 strain

| Antibiotics[#] | murepavadin | ciprofloxacin |
|---|---|---|
| MIC | 0.0625 | 0.25 |

[#] | Data represent the results of three independent experiments.

**Table S3.** Clinical isolates used in this study

| Strain[#] | Isolation sites | MIC (mg/L) | |
|---|---|---|---|
| | | Mur[a] | Cip[a] |
| CI-PA-25 | Sputum | 0.0625 | 0.25 |
| CI-PA-26 | Sputum | 0.03125 | 0.125 |
| CI-PA-42 | Urine | 0.03125 | 0.25 |
| CI-PA-45 | Urine | 0.03125 | 0.25 |
| CI-PA-92 | Sputum | 0.03125 | 0.25 |
| CI-PA-82 | Bronchoalveolar lavage fluid | 0.03125 | 0.125 |
| CI-PA-83 | Sputum | 0.0625 | 0.25 |
| CI-PA-123 | Diabetic foot | 0.0625 | 0.25 |
| CI-PA-128 | Diabetic foot | 0.03125 | 0.125 |
| CI-PA-132 | Diabetic foot | 0.0625 | 0.125 |
| CI-PA-161 | Diabetic foot | 0.0625 | 0.125 |
| CI-PA-139 | Diabetic foot | 0.03125 | 0.25 |

[#] | Data represent the results of three independent experiments. Strains isolated from the same hospital were grouped by the same color.

[a] | Mur, murepavadin; Cip, ciprofloxacin.
